# Patulin Occurrence in Traditional Turkish Molasses (Pekmez): Comparisons Across Product Types and Associations with pH and Soluble Solids

**DOI:** 10.3390/foods15213798

**Published:** 2026-10-23

**Authors:** Nesrin İçli, Sümeyye Begüm Atalan, Funda Işık, Kadriye Elif İmre

**Affiliations:** Department of Nutrition and Dietetics, Faculty of Health Sciences, Kastamonu University, 37150 Kastamonu, Türkiye; begumatalan@kastamonu.edu.tr (S.B.A.); fisik@kastamonu.edu.tr (F.I.); keimre@kastamonu.edu.tr (K.E.İ.)

**Keywords:** mycotoxins, HPLC-DAD, dietary exposure, food safety, hazard quotient

## Abstract

Patulin occurrence in traditional Turkish molasses (pekmez), particularly products made from raw materials other than apple, remains poorly characterized. This exploratory cross-sectional study determined patulin concentrations, pH, and soluble-solids content (°Brix) in 25 purposively selected samples obtained from selected provinces in Türkiye, comprising apple (*n* = 6), mulberry (*n* = 7), sugar beet (*n* = 6), and grape (*n* = 6) molasses. Patulin was quantified by high-performance liquid chromatography with diode-array detection (HPLC-DAD), and between-type differences and associations with pH and °Brix were evaluated using nonparametric methods. The median patulin concentration was 67.30 µg/kg (interquartile range: 39.75–107.65 µg/kg; range: below the limit of detection to 672.50 µg/kg). No statistically significant differences in patulin concentrations or °Brix were detected among molasses types (*p* = 0.442 and *p* = 0.108), whereas pH differed significantly (*p* = 0.001), with apple molasses having a lower pH than mulberry and grape molasses. No statistically significant correlations were detected between patulin concentration and either pH or °Brix. Fifteen of the 25 analyzed samples (60.0%) had concentrations above the 50 µg/kg contextual comparison value, which is not a product-specific regulatory limit for molasses. In serving-based adult exposure scenarios, median-based hazard quotients ranged from 0.041 to 0.084, while the maximum-concentration scenario yielded 0.648. Without matrix-specific recovery and precision, non-apple comparisons remain preliminary. These findings provide initial comparative evidence of patulin occurrence within the analyzed sample set and should not be generalized to traditional molasses production throughout Türkiye. Broader surveillance should incorporate geographically representative sampling and habitual-consumption data.

## 1. Introduction

Traditional Turkish molasses (pekmez) is a concentrated product prepared in Türkiye from sugar-rich raw materials, including grape, mulberry, fig, apple, and sugar beet. It is produced in both liquid and solid forms; traditional liquid molasses processing generally involves selecting and washing the raw material, crushing and pressing it, and concentrating the resulting liquid by boiling, although the process differs for mulberry molasses [1]. Traditional molasses processing may also include a clarification step before concentration. In grape molasses production, lime-rich pekmez earth is added to the pressed must to facilitate clarification and filtration and to reduce acidity; after settling, the clarified fraction is separated and concentrated by boiling [2]. The use of different raw materials is reflected in variation among the resulting products. In a recent comparison of eight traditionally produced molasses varieties, total soluble solids ranged from 55.36% to 80.92% [3]. Similarly, an analysis of seven commercial molasses types revealed wide ranges in glucose, fructose, organic acid, total phenolic, and moisture contents [4].

Patulin is a secondary metabolite produced by several fungal species, particularly members of the genera *Aspergillus*, *Byssochlamys*, and *Penicillium*, with *Penicillium expansum* recognized as the principal producer associated with apples and apple-derived products [5]. Although apples remain its best-established source, analytical surveys indicate that patulin occurrence is not restricted to apple matrices. Hussain et al. analyzed 381 samples of apples, grapes, and their derived products by high-performance liquid chromatography with ultraviolet detection (HPLC-UV) and detected patulin in both fruit groups and their processed products; among the 172 grape and grape-juice samples, 112 were positive [6]. In a later study of 442 fruit samples collected at farm, transport, and market stages, patulin-positive samples were reported for oranges, apples, apricots, lemons, and guava [7]. An untargeted liquid chromatography–mass spectrometry (LC–MS) metabolomic study also reported patulin in some sugar beet root samples collected during prolonged post-harvest storage [8]. Patulin occurs mainly in mold-damaged fruit; however, internal fungal growth following insect damage or other tissue invasion may also result in contamination of fruit that appears externally sound [9]. Fruit condition at harvest, bruising or other physical damage during handling, and storage conditions that permit mold growth can influence the likelihood of contamination [9,10]. Processing does not necessarily eliminate patulin already present in fruit juice. In a pilot-scale study of cloudy apple juice, centrifugation and pasteurization did not reduce patulin concentrations [11].

Patulin is of toxicological importance because various adverse effects, including hepatic and intestinal injury, immunotoxicity, genotoxicity, and neuronal cell damage, have been reported primarily in experimental animal and cell models [12,13,14,15,16]. Based on its toxicological evaluation, the Joint FAO/WHO Expert Committee on Food Additives (JECFA) established a provisional maximum tolerable daily intake (PMTDI) of 0.4 µg/kg body weight per day for patulin [17]. In Türkiye, the Turkish Food Codex specifies a maximum patulin level of 50 µg/kg for fruit juices, fruit juices reconstituted from concentrate, concentrated fruit juice, and fruit nectars. For concentrated fruit juice, this limit applies to the reconstituted product [18]. Although traditional molasses is also produced by concentrating the extracted juice through boiling [1], it is not listed as a separate product category with a specific maximum patulin level [18].

These toxicological and regulatory considerations make the potential occurrence of patulin in molasses relevant to food safety. This issue is particularly important because molasses is a traditional food in Türkiye and is commonly produced using traditional methods [1,3]. The use of mold-damaged raw materials, inadequate hygiene during handling, and storage conditions conducive to fungal growth may increase the likelihood of patulin contamination [9,10]. Laboratory experiments have shown that temperature, pH, and water activity influence *P. expansum* growth and patulin production, and that conditions favoring fungal growth do not necessarily maximize toxin production [19]. pH is relevant to patulin assessment because *P. expansum* can acidify colonized tissue through gluconic acid production, and this dynamic acidification is associated with patulin biosynthesis and accumulation; nevertheless, low pH alone does not determine patulin accumulation [20]. In molasses, °Brix expresses total soluble solids, and considerable variation in °Brix values has been reported among products made from different raw materials [3]. Published evidence for patulin in traditional concentrated products remains scarce. Patulin has been reported in apple sour, a related traditional concentrated apple product [21], while only one previous study has reported patulin in traditionally produced apple molasses [22]. To the best of our knowledge, no previous study has compared patulin occurrence in apple, mulberry, sugar beet, and grape molasses within a single research design. Therefore, this exploratory study aimed to determine patulin concentrations in traditionally produced apple, mulberry, sugar beet, and grape molasses, compare patulin concentrations, pH, and °Brix among product types, and examine the associations between patulin concentrations and pH and °Brix.

## 2. Materials and Methods

### 2.1. Study Design and Sample Collection

A cross-sectional exploratory study was conducted to evaluate patulin occurrence and selected physicochemical characteristics in homemade Turkish molasses. The study included 25 independent samples representing four molasses types: apple (*n* = 6), mulberry (*n* = 7), sugar beet (*n* = 6), and grape (*n* = 6). Samples were purposively selected from accessible household producers and sales points offering homemade regional products. Each sample originated from a different producer and was considered an independent sampling unit.

Apple and sugar beet molasses samples were obtained from Kastamonu Province, grape molasses samples from Aksaray and Nevşehir Provinces, and mulberry molasses samples from Bartın and Giresun Provinces. According to information provided by the producers and sellers, the samples had reportedly been produced in 2021. Before collection, the products had reportedly been stored in large plastic containers or glass jars in cool, dry storage areas or on shelves. All products were unpackaged and supplied in bulk. Following collection, the samples were transferred into clean glass containers, protected from light, and stored at 4 °C in the cold storage facility of the Central Research Laboratory (MERLAB) at Kastamonu University until analysis. Sample collection was completed over approximately six months, and laboratory analyses were conducted after the final sample had been obtained. Because the samples were acquired at different times during the collection period, their storage durations before analysis were not identical. Exact production dates, individual collection dates, and product-specific storage durations before analysis were not available.

Each sample was measured in duplicate for pH, soluble-solids content (°Brix), and patulin concentration. The arithmetic mean of the two technical measurements was used as the sample-level value in all statistical analyses.

### 2.2. Chemicals, Reagents, and Standards

Glacial acetic acid, anhydrous sodium sulfate, and reagent-grade sodium carbonate were supplied by Merck (Darmstadt, Germany). Acetonitrile and ethyl acetate of HPLC grade, together with an aqueous pectinase preparation with a typical activity of at least 3800 units/mL, were purchased from Sigma-Aldrich (St. Louis, MO, USA). Ultrapure water was obtained from a Human Power 2 purification system (Seoul, Republic of Korea). When necessary, glacial acetic acid was used to adjust the water to pH 4.0.

A certified patulin standard containing 100 µg/mL patulin in acetonitrile was obtained from Sigma-Aldrich. To prepare the intermediate standard, a 200 µL aliquot of the certified standard solution was transferred into a 10 mL volumetric flask. Acetonitrile was removed under a nitrogen stream at room temperature, after which the residue was immediately brought to volume with ultrapure water adjusted to pH 4.0. The resulting intermediate solution contained 2.0 µg/mL patulin. Calibration standards were obtained by diluting this solution with pH-adjusted ultrapure water.

### 2.3. Determination of pH and Soluble-Solids Content

Sample pH was measured potentiometrically in accordance with TS 1728 ISO 1842 [23]. Soluble-solids content was determined using the refractometric procedure specified in TS 4890 [24] and reported as °Brix. Both measurements were conducted at 20 °C. Two technical determinations were performed for each parameter in every sample.

### 2.4. Patulin Extraction and Clean-Up

The extraction procedure was based on EN 14177 [25] and previously reported reversed-phase high-performance liquid chromatography with diode-array detection (HPLC-DAD) procedures for patulin analysis in fruit-derived matrices [21,26], with modifications appropriate for the molasses samples.

For each independent extraction, 5 g of homogenized molasses was transferred into a 50 mL tube and mixed with 5 mL of ethyl acetate for 1 min using a VELP Scientifica Classic vortex mixer (Milan, Italy). Subsequently, 70 µL of pectinase preparation and 2 mL of ultrapure water adjusted to pH 4.0 were introduced into the tube. After an additional 1 min of vortex mixing, the mixture was kept at room temperature for 2 h. Phase separation was then achieved by centrifugation at 5000 rpm for 5 min, and the liquid phase was transferred into a clean tube.

The remaining sample material underwent a second extraction with another 5 mL portion of ethyl acetate. Following 1 min of vortex mixing and centrifugation, the liquid phases obtained from both extraction steps were pooled. The combined extract was treated with 1 mL of 1.5% (*w*/*v*) aqueous sodium carbonate, vortex-mixed for 1 min, and centrifuged. The washing step was completed within 1–2 min, and the upper organic layer was collected without delay. The residual carbonate phase was subsequently mixed with an additional 5 mL of ethyl acetate for 1 min. After centrifugation, the resulting organic layer was recovered immediately and added to the previously collected extract.

Residual moisture was removed by adding 1.25 g of anhydrous sodium sulfate to the pooled organic extract. After vortex mixing, the mixture was passed through filter paper into an evaporation flask. The sodium sulfate and filter paper were rinsed with 2 mL of ethyl acetate, and this rinse was added to the filtrate.

The combined filtrate was evaporated to dryness at 40 °C under nitrogen in a water bath. The residue was transferred into an HPLC vial using 1 mL of ethyl acetate to rinse the evaporation flask. Following a second evaporation to dryness at 40 °C under nitrogen, the vial residue was promptly reconstituted in 1 mL of filtered ultrapure water adjusted to pH 4.0 using acetic acid. The prepared extracts were maintained at −18 °C until HPLC-DAD measurement. Two independent extractions were performed for each molasses sample.

### 2.5. HPLC-DAD Determination of Patulin

Chromatographic determination of patulin was carried out using a Shimadzu LC-20A Prominence high-performance liquid chromatography system coupled to a diode-array detector (Shimadzu Corporation, Kyoto, Japan), using an approach based on previously published methods for patulin determination in fruit-derived matrices [21,26] and the analytical principles described in EN 14177:2003 [25], with modifications for molasses samples.

Separation was performed on an Inertsil ODS-3 C18 column supplied by GL Sciences (Tokyo, Japan), with dimensions of 250 × 4.6 mm and a particle size of 5 µm. The isocratic mobile phase contained pH 4.0 ultrapure water and acetonitrile in a 97:3 volume ratio. It was delivered at 1.5 mL/min while the column was maintained at 25 °C. Each injection had a volume of 50 µL, and diode-array detection was conducted at 276 nm.

Patulin peaks in the molasses extracts were assigned based on agreement of their retention times with those of the patulin reference standard analyzed under identical chromatographic conditions. Under the applied chromatographic conditions, 5-hydroxymethylfurfural (HMF) and patulin eluted at approximately 6.35 and 8.28 min, respectively. Their chromatographic separation was evaluated using a mixed standard solution containing 5 µg/mL HMF and 0.050 µg/mL patulin (Supplementary Figure S2). Representative chromatograms of the apple-derived matrix blank and a separately prepared fortified matrix used to illustrate the chromatographic separation of HMF and patulin are presented in Supplementary Figure S3. Representative chromatograms of patulin-containing samples from apple, mulberry, sugar beet, and grape molasses, including the sample with the highest measured concentration, are provided in Supplementary Figure S4.

DAD spectral-comparison and peak-purity outputs were not available for retrospective evaluation. Therefore, peak identity was not considered to have been confirmed by spectral matching, and the available chromatographic evidence does not exclude all possible matrix-specific co-elution. This limitation was considered when interpreting the quantitative results.

Calibration-standard runs lasted 11 min, whereas the run time for sample extracts was extended to 55 min to prevent late-eluting matrix constituents from carrying over into subsequent analyses.

Because molasses is a concentrated and viscous product, the sample aliquots were weighed rather than volumetrically measured, and concentrations measured in the final extracts were converted to sample-level concentrations using the following equation:

Sample concentration (µg/kg) = [extract concentration (µg/mL) × final reconstitution volume (mL) × dilution factor]/sample mass (kg)

The final reconstitution volume was 1 mL, and the initial sample mass was 0.005 kg. For undiluted extracts, the resulting conversion factor was 200. For the single extract whose initial concentration exceeded the upper calibration limit of 2.000 µg/mL, an aliquot was diluted 1:1 (*v*/*v*) with filtered ultrapure water adjusted to pH 4.0 using glacial acetic acid and reanalyzed. A dilution factor of 2 was applied when calculating the final sample concentration. Final concentrations were expressed as µg/kg of molasses.

### 2.6. Calibration, Method Performance, and Analytical Quality Assurance

**Figure 1:**
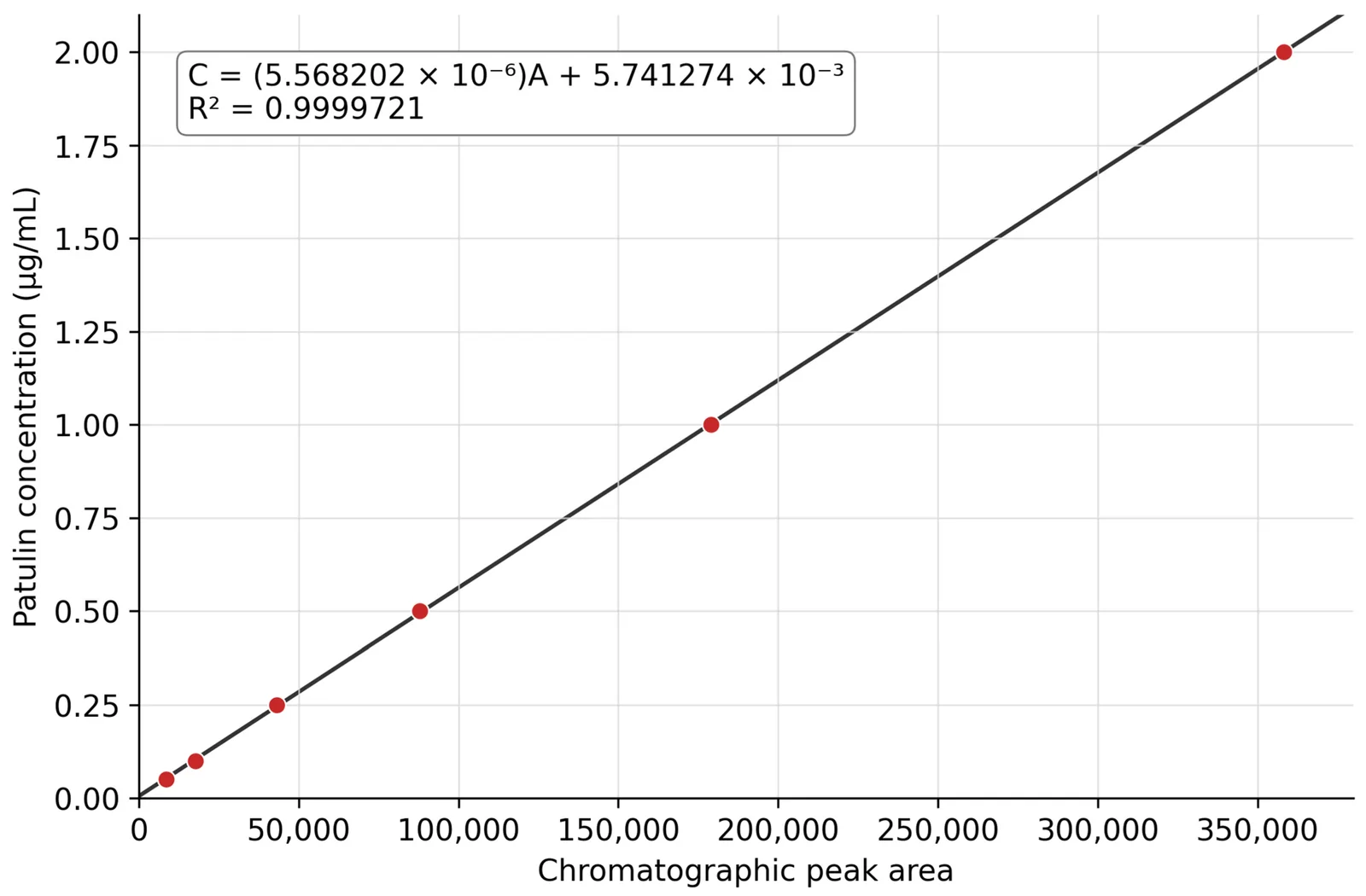
External-standard calibration curve for the quantification of patulin by HPLC-DAD. Six calibration levels ranging from 0.050 to 2.000 µg/mL were fitted using unweighted linear regression with a non-zero intercept.

The extraction and HPLC-DAD procedure was adapted from the method previously described by İçli [21], with modifications for the molasses matrix, as described in Section 2.4 and Section 2.5. Patulin was quantified by external-standard calibration using six concentration levels prepared in ultrapure water adjusted to pH 4.0: 0.050, 0.100, 0.250, 0.500, 1.000, and 2.000 µg/mL. The calibration data were fitted using an unweighted linear regression model with the intercept retained. The following calibration equation was obtained:

C = (5.568202 × 10^−6^)A + 5.741274 × 10^−3^

where *C* represents the patulin concentration (µg/mL) and *A* represents the chromatographic peak area. The calibration model yielded a coefficient of determination of *R*^2^ = 0.9999721. The mean concentration-to-peak-area ratio was 5.698009 × 10^−6^, with a standard deviation of 1.041549 × 10^−7^ and a relative standard deviation (RSD) of 1.83%. The calibration curve is presented in Figure 1, and the overlaid chromatograms of the six calibration standards are provided in Supplementary Figure S1. A single response was available at each calibration level; therefore, replicate-calibration statistics could not be calculated. The back-calculated concentrations differed from the nominal concentrations by −1.77% to 6.99%. Examination of the calibration residuals showed no evident concentration-dependent pattern; therefore, the unweighted linear regression model was retained. However, because replicate responses were unavailable at each calibration level, heteroscedasticity could not be formally assessed and alternative weighting schemes could not be compared. The individual calibration responses, back-calculated concentrations, residuals, and relative errors are presented in Table S1.

The solution-level limits of detection (LOD) and quantification (LOQ), determined using signal-to-noise criteria of 3 and 10, respectively, were 0.0146 and 0.0441 µg/mL. Based on the initial sample mass of 5 g and final reconstitution volume of 1 mL, the corresponding sample-level LOD and LOQ for undiluted extracts, calculated from the unrounded solution-level values, were 2.91 and 8.83 µg/kg, respectively. The lowest calibration level of 0.050 µg/mL corresponded to 10.0 µg/kg of molasses. One sample result was below the sample-level LOD, and two were between the sample-level LOD and LOQ; all three results were also below the sample-equivalent lowest calibration level. These observations were therefore treated as left-censored rather than as exact quantified concentrations.

Recovery and precision were evaluated within the present study. To prepare a blank matrix for recovery and repeatability studies, fruit juice was obtained by pressing fresh, undamaged apples. Subsequently, the juice underwent the traditional molasses-production process, including clarification with marl (locally known as “pekmez soil,” a calcium carbonate-rich material traditionally used to reduce acidity and facilitate clarification during molasses production). For each fortification level, 5 g of the blank apple-derived molasses matrix was fortified with 1 mL of an aqueous patulin working solution at 0.15 or 1.5 µg/mL and homogenized before undergoing the complete extraction procedure. These additions corresponded to sample-equivalent fortification levels of 30 and 300 µg/kg, respectively. Because the final extract was reconstituted to 1 mL and the blank matrix contained no detectable patulin, the corresponding target concentrations in the final extracts were 0.15 and 1.5 µg/mL, respectively. Six independently prepared replicates at each fortification level were subjected to the complete analytical procedure.

At the sample-equivalent fortification levels of 30 and 300 µg/kg, the mean recoveries were 105.4% and 102.7%, the intra-day relative standard deviations (RSDs) were 4.9% and 1.2%, and the inter-day RSDs were 5.3% and 3.8%, respectively. These results were within the general recovery range of 70–120% and the precision limit of 20% specified in Commission Implementing Regulation (EU) 2023/2782 for confirmatory methods used in mycotoxin analysis [27]. This comparison relates only to the reported recovery and precision parameters and does not, by itself, establish compliance with all performance requirements for a confirmatory method. These performance estimates apply only to the two fortification levels evaluated in the apple-derived molasses matrix and should not be extrapolated to other concentration levels or molasses matrices.

The same ethyl-acetate extraction, carbonate clean-up, and HPLC-DAD procedure was applied to all molasses samples to maintain consistent analytical handling. However, applying the same procedure does not establish equivalent recovery or analytical response across different matrices. No matrix-specific recovery or precision studies were performed for mulberry, grape, or sugar beet molasses, and matrix effects were not independently assessed using matrix-matched calibration or standard addition. Accordingly, the apple-molasses performance data do not establish equivalent quantitative performance across all four matrices, and the quantitative results for mulberry, grape, and sugar beet molasses should be interpreted as preliminary estimates.

Analytical quality assurance during the study was monitored by reanalyzing a mid-level calibration standard and a designated study sample (Sample 1) after every 10 sample analyses. Because no certified reference material was available specifically for patulin in molasses, surplus Food Analysis Performance Assessment Scheme (FAPAS) apple juice proficiency-test material (Proficiency Test 1632 017) was used as an external quality-control material. Six independent preparations of the material were analyzed. The assigned and measured concentrations were retained in µg/L, consistent with the volume-based unit specified for the apple-juice test material. Recovery was calculated as the measured concentration divided by the assigned concentration and multiplied by 100. The analytical performance obtained for the FAPAS material is reported in Section 3.1, and the results for the six individual preparations are provided in Table 1. The FAPAS material was used solely for external analytical quality control and did not constitute matrix-specific validation for any of the molasses types.

### 2.7. Contextual Comparison Value

A patulin concentration of 50 µg/kg was used solely as a contextual comparison value for descriptive purposes. This value corresponds to the maximum level established for specified fruit-juice categories under the Turkish Food Codex Contaminants Regulation and the corresponding European Union framework [18,28]. No product-specific maximum level for patulin in traditional molasses was identified in these regulations; therefore, 50 µg/kg was not interpreted as a legal maximum level or a product-specific safety threshold for the molasses samples. The relevance of this value to sugar beet molasses is more limited because sugar beet is not a fruit-derived raw material and the regulatory value was not established for sugar beet products. Nevertheless, the same value was applied to all four molasses groups solely to provide a uniform descriptive reference; this does not imply that the fruit-juice maximum level is legally applicable to sugar beet molasses. In addition, the Turkish regulation states that, where a specific maximum level is not established for a processed, dried, diluted, or multi-ingredient food, changes arising from processing or concentration/dilution should be taken into account [18].

### 2.8. Screening-Level Dietary Exposure Assessment

A deterministic screening-level dietary exposure assessment was conducted for adult consumers. In the absence of nationally representative data on habitual homemade molasses consumption, a daily intake of 27 g was assumed, corresponding to one standard serving of molasses specified in the Türkiye Beslenme Rehberi (TÜBER) 2022 [29]. This amount was used as a serving-based scenario and should not be interpreted as the mean daily consumption of the Turkish population. A default adult body weight of 70 kg was used in accordance with the guidance of the European Food Safety Authority [30]. The assessment was restricted to adults because reliable age-specific data on habitual molasses consumption among children were unavailable. Constructing a child-exposure scenario without such data would have required additional assumptions regarding consumption and body weight. Therefore, the resulting estimates apply only to the specified adult scenario and should not be extrapolated to children.

Estimated daily intake (EDI) was calculated using the following equation:

$$\mathrm{EDI}=\frac{C\times \mathrm{IR}}{\mathrm{BW}}$$

where *C* is the patulin concentration in molasses (µg/kg), *IR* is the assumed daily molasses intake (0.027 kg/day), and *BW* is adult body weight (70 kg). Calculations were performed using the overall median, molasses-type-specific median, and maximum observed patulin concentrations. The maximum observed concentration was used to construct a conservative high-concentration scenario and was not intended to represent typical consumer exposure.

Risk characterization was performed by comparing the EDI with the provisional maximum tolerable daily intake (PMTDI) of 0.4 µg/kg body weight/day established for patulin by the Joint FAO/WHO Expert Committee on Food Additives [17]. The hazard quotient (HQ) was calculated as:

$$\mathrm{HQ}=\frac{\mathrm{EDI}}{\mathrm{PMTDI}}$$

An HQ below 1 indicated that the estimated exposure did not exceed the PMTDI, whereas an HQ above 1 indicated an exceedance of the health-based guidance value. The percentage contribution to the PMTDI was calculated as HQ × 100. Because the assumed intake was based on a standard serving rather than habitual consumption data, the estimates were interpreted as serving-based screening scenarios rather than population-level dietary exposure estimates. The assessment considered exposure from molasses alone and did not include other dietary sources of patulin.

### 2.9. Statistical Analysis

For each sample, the arithmetic mean of the two technical measurements was calculated for pH, °Brix, and patulin concentration, and each sample was considered an independent unit of analysis. Continuous variables were summarized as median with first and third quartiles (Q1–Q3) and minimum–maximum values.

The distribution of each variable within each molasses group was examined graphically and assessed using the Shapiro–Wilk test. Homogeneity of variances among molasses groups was evaluated using the median-centered Levene test (Brown–Forsythe modification). Because the normality assumption was not consistently satisfied and the group sample sizes were small, nonparametric methods were used for the primary analyses.

Differences in pH, °Brix, and patulin concentrations among molasses types were evaluated using the Kruskal–Wallis H test. When the omnibus test was statistically significant, pairwise comparisons were performed using Dunn’s test with Holm adjustment for multiple comparisons. Epsilon-squared (ε^2^) was calculated as the effect-size measure for the Kruskal–Wallis test. Associations between patulin concentration and pH or °Brix were examined using Spearman’s rank correlation analysis.

For the statistical analysis of left-censored patulin results, the single result below the sample-level LOD was replaced with LOD/2, whereas the two results between the LOD and LOQ were replaced with LOQ/2. Using the unrounded sample-level analytical limits, the substituted values used in the primary analysis were 1.4563 and 4.4132 µg/kg, respectively. Robustness was assessed using lower- and upper-bound sensitivity analyses. In the lower-bound analysis, the result below the LOD was assigned a value of zero and the results between the LOD and LOQ were assigned the LOD. In the upper-bound analysis, these results were assigned the LOD and LOQ, respectively. Patulin-related descriptive statistics, group comparisons, correlations, proportions above the 50 µg/kg contextual comparison value, and exposure estimates were recalculated under both sensitivity scenarios. Unless otherwise stated, the reported results were based on the primary substitution approach.

The numbers and proportions of samples with patulin concentrations above the 50 µg/kg contextual comparison value were calculated for each molasses type and for the overall sample. Exact 95% Clopper–Pearson confidence intervals were calculated for these proportions. Differences among molasses types in the proportions above this contextual comparison value were evaluated using the Fisher–Freeman–Halton exact test because of the small expected cell frequencies.

All statistical tests were two-sided, and *p*-values < 0.05 were considered statistically significant. Statistical analyses and graphical presentations were performed using Python version 3.14.7 with pandas version 3.0.5, NumPy version 2.5.2, SciPy version 1.18.1, Matplotlib version 3.11.1, and seaborn version 0.13.2. ChatGPT-5.6 Sol (OpenAI, San Francisco, CA, USA; accessed October 2026) was used to assist with the development and debugging of the Python code employed in the statistical analyses. The authors independently determined the analytical procedures, executed and reviewed the code, verified the outputs against the source data, and interpreted the findings.

## 3. Results and Discussion

### 3.1. Analytical Quality-Control Performance

The assigned patulin concentration of the FAPAS surplus test material was 37.5 µg/L. Analysis of six independent preparations yielded a mean patulin concentration of 40.17 ± 0.65 µg/L, corresponding to a mean recovery of 107.13 ± 1.73% and a relative standard deviation (RSD) of 1.61% (Table 1). These results showed close agreement among six independent preparations.

Because the FAPAS surplus material consisted of apple juice and was analyzed outside the original proficiency-testing round, the findings were used solely for analytical quality-control purposes and were not interpreted as a formal proficiency-test result or as matrix-specific validation for any of the molasses types.

### 3.2. Distribution of Patulin in Molasses Samples

**Figure 2:**
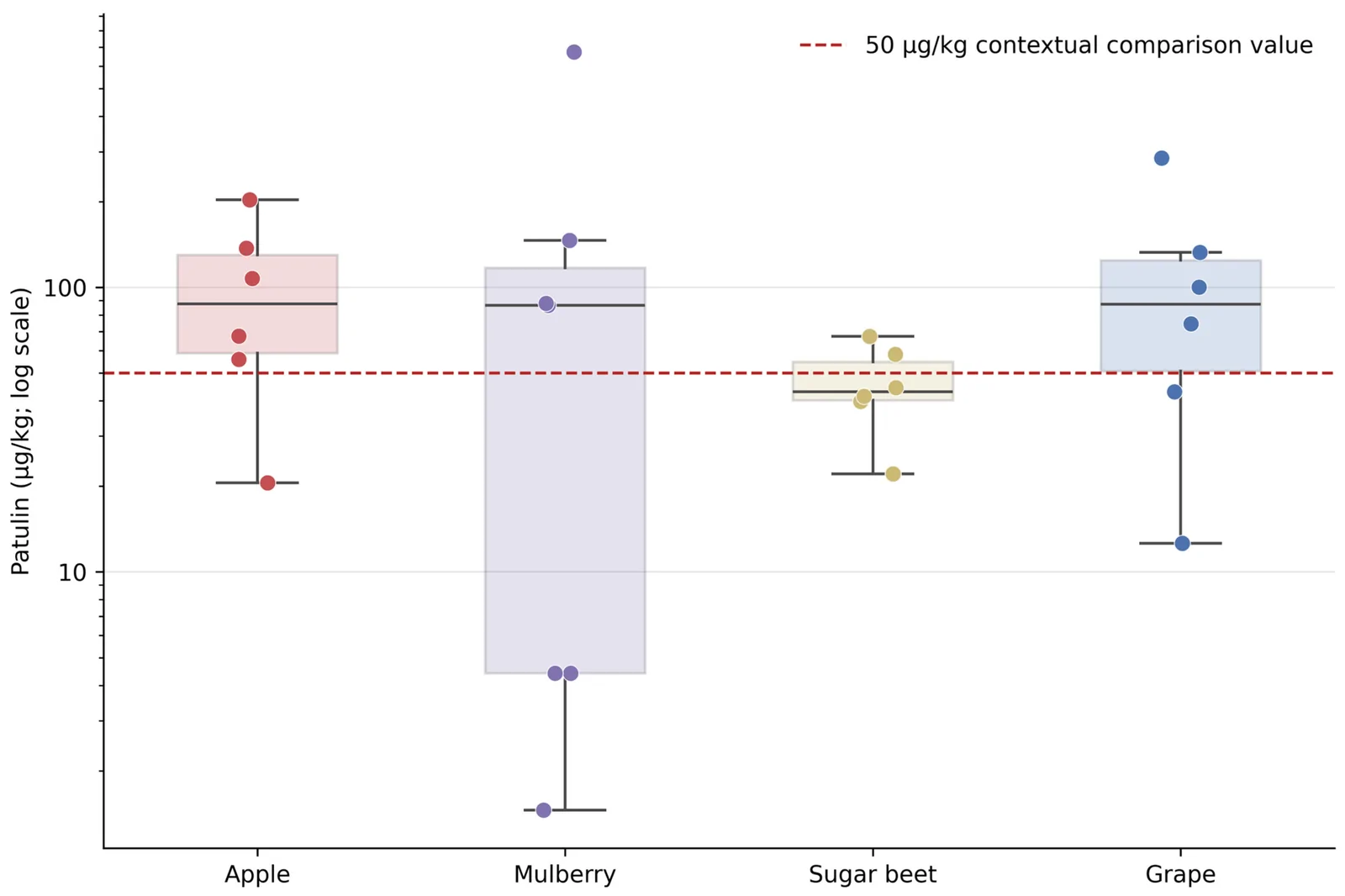
Distribution of patulin concentrations by molasses type. Points represent individual samples, boxes indicate the median and interquartile range (Q1–Q3), and whiskers extend to the most extreme values within 1.5 times the interquartile range. For visualization, the result below the LOD was plotted at LOD/2, and the two results between the LOD and LOQ were plotted at LOQ/2. The *y*-axis is presented on a logarithmic scale. The red dashed line indicates the 50 µg/kg contextual comparison value, which should not be interpreted as a product-specific regulatory limit for molasses. LOD, limit of detection; LOQ, limit of quantification.

A total of 25 molasses samples were analyzed, including six apple, seven mulberry, six sugar beet, and six grape molasses samples. One mulberry molasses result was below the sample-level LOD, and two mulberry molasses results were between the LOD and LOQ. After applying the substitution approach described in Section 2.9, the overall median patulin concentration was 67.30 µg/kg (Q1–Q3: 39.75–107.65 µg/kg). Sample results ranged from below the LOD to 672.50 µg/kg. The maximum individual concentration (672.50 µg/kg) was observed in one mulberry molasses sample; this observation should not be interpreted as indicating that the mulberry molasses group had the highest typical patulin concentrations. The physicochemical characteristics and patulin concentrations according to molasses type are presented in Table 2, while the distribution of individual sample-level patulin concentrations is shown in Figure 2.

For apple molasses, the median patulin concentration of 87.58 µg/kg was within the ranges reported for two traditional apple products. İçli (2019) detected patulin in 39 apple sour samples at concentrations ranging from 20 to 1416 µg/kg, with a mean of 284 ± 307 µg/kg [21]. Şen (2023) reported concentrations of 22–371 µg/kg and a mean of 139 ± 106 µg/kg in 26 traditionally produced apple molasses samples [22]. Hussain et al. (2020) also detected patulin in both fresh grapes and grape juice; however, these matrices are not directly equivalent to concentrated grape molasses and were therefore not used as quantitative benchmarks for the present grape molasses results [6]. In the sources examined, directly comparable patulin measurements were not identified for mulberry or sugar beet molasses.

### 3.3. Comparisons Among Molasses Types

No statistically significant difference in patulin concentrations was detected among the four molasses types (Kruskal–Wallis H(3) = 2.69, *p* = 0.442, ε^2^ = 0.00). The lower- and upper-bound sensitivity analyses yielded the same statistical conclusion. Similarly, no statistically significant difference was detected in °Brix among molasses types (H(3) = 6.08, *p* = 0.108, ε^2^ = 0.15). In contrast, pH differed significantly among molasses types (H(3) = 15.79, *p* = 0.001, ε^2^ = 0.61) (Table 2).

Dunn’s test with Holm adjustment showed that the pH of apple molasses was significantly lower than that of mulberry molasses (adjusted *p* = 0.037) and grape molasses (adjusted *p* < 0.001). The remaining pairwise comparisons were not statistically significant (all adjusted *p* > 0.05). The between-group comparisons should be interpreted in light of the geographical distribution of the samples. Apple and sugar beet molasses were obtained from Kastamonu, whereas grape and mulberry molasses were collected from other provinces. Product type and geographical origin were therefore partly confounded, and the contribution of raw-material type could not be separated from possible regional influences or differences in raw-material characteristics, pre-collection storage conditions, and production practices. Accordingly, the lower pH observed in apple molasses cannot be attributed solely to raw-material type. Similarly, the absence of statistically significant differences in patulin concentrations and °Brix should be interpreted cautiously in light of the partial confounding between product type and geographical origin. The small group sizes (6–7 samples per product type) limit the statistical power of the between-group comparisons. Patulin concentrations showed considerable within-group variability, including a maximum concentration of 672.50 µg/kg in a mulberry molasses sample. The near-zero effect-size estimate for patulin (ε^2^ = 0.00) should therefore be interpreted cautiously in view of the small sample size and heterogeneous concentration distributions and should not be taken as conclusive evidence of negligible between-type differences. No statistically significant differences in patulin concentrations or °Brix were detected in this sample set; these results do not establish equivalence in either variable across molasses types.

Direct comparison with previous studies is difficult because comparable evidence from non-apple matrices remains scarce. Among the limited available studies, Ostry et al. (2018) detected patulin in a proportion of grape must samples obtained from grapes naturally contaminated with *P. expansum* [31]. Majumdar et al. (2024) also reported patulin in some sugar beet root samples examined during post-harvest storage using untargeted metabolomic analysis, although no meaningful correlation was identified between patulin and the associated fungal genera [8]. In the literature reviewed, no directly comparable targeted patulin study was identified for mulberry or mulberry molasses. Patulin is a secondary metabolite produced by several fungal species [5], and thermal processing may not completely eliminate patulin already present [11]. On the basis of the cited literature, carry-over from contaminated raw materials is one possible explanation for the patulin detected in the finished molasses. This hypothesis could not be tested because only finished products were analyzed; therefore, the source and timing of contamination cannot be established from the present data. External appearance alone also does not exclude patulin contamination. The Codex Alimentarius Commission (2003) notes that internally mold-affected fruit may appear externally undamaged [9]. In a farm-to-fork risk assessment model, Baert et al. (2012) reported that the interval between apple delivery and processing affected predicted patulin concentrations, while sorting visibly infected apples reduced the predicted levels in apple juice [32]. Coton et al. (2020) found that patulin was concentrated primarily within mold lesions in artificially inoculated apples but could also be detected in the surrounding tissue [33]. Aroud et al. (2021) further showed that the pilot-scale apple juice processing steps examined did not completely remove patulin [11]. Together, these apple-based findings suggest that raw-material quality, sorting, storage before processing, and processing conditions may be relevant to patulin occurrence in molasses. Their contributions to the concentrations observed in the present samples remain untested because these factors were not assessed for the individual samples. Findings from apple or apple juice systems also cannot be directly generalized to molasses produced from different raw materials.

No statistically significant between-type difference in °Brix was detected in the analyzed sample set. Traditional molasses production generally involves boiling the extract until the desired consistency is reached [1]. Dilek et al. (2025) reported that total soluble solids ranged from 55.36% to 80.92% across eight traditionally produced molasses varieties [3]. One possible explanation for the overlapping °Brix ranges observed here is concentration to broadly similar final consistencies during production. However, this hypothesis could not be tested because boiling durations and the criteria used to determine the processing endpoint were not recorded for the individual samples.

The lower pH of apple molasses compared with mulberry and grape molasses is consistent with previous findings on sour apple molasses. Koca and Karadeniz (2009) reported total acidity values ranging from 9.90 to 75.30 g/kg in sour apple molasses and noted that acidity is not neutralized using pekmez earth or CaCO_3_ during its production [34]. Kuşçu and Bulantekin (2016) found malic acid to be the predominant organic acid in the apple examined in their study and, citing previous research, noted that CaCO_3_ can reduce acidity by precipitating malic and tartaric acids as calcium salts [35]. These literature findings suggest organic-acid composition and acidity-neutralization practices as possible explanations for the lower pH observed in the apple molasses group. Nevertheless, neither organic-acid profiles nor the use of neutralizing agents was assessed in the present samples. In addition, product type was partly confounded with geographical origin. Therefore, the mechanism underlying the between-type difference in pH cannot be established from the current data.

When evaluated by product type, the agreement between the pH and °Brix ranges observed in the present study and those reported in the literature varied. The apple molasses ranges (pH 3.27–3.87; °Brix 58.5–73.0) overlapped with the corresponding ranges reported by Koca and Karadeniz (2009): pH 3.04–4.58 and °Brix 65–85 [34]. For mulberry molasses, the °Brix range (60.5–80.0) partially overlapped the water-soluble dry matter range reported by Salık et al. (2021) (70.60–83.80%), whereas the pH range (3.74–4.71) was lower than their reported range (4.86–5.17) [36]. The pH and °Brix ranges observed for grape and sugar beet molasses showed partial to substantial overlap with the corresponding published values [37,38]. Overall, agreement with the published literature was more apparent for °Brix, whereas pH ranges showed greater variation among studies. Because the studies involved different sample sets and production conditions, these comparisons should be considered descriptive, and overlap between ranges should not be interpreted as evidence of statistical equivalence.

### 3.4. Samples Relative to the 50 µg/kg Contextual Comparison Value

Fifteen of the 25 molasses samples (60.0%) had patulin concentrations above the 50 µg/kg contextual comparison value. The highest proportion was observed in apple molasses (83.3%), whereas the lowest proportion was observed in sugar beet molasses (33.3%) (Table 3). No statistically significant difference in these proportions was detected among the molasses types (Fisher–Freeman–Halton exact test, *p* = 0.429). The classification of samples relative to the contextual comparison value and the corresponding proportions remained unchanged across the primary, lower-bound, and upper-bound analyses.

The Turkish Food Codex Contaminants Regulation specifies a maximum patulin level of 50 µg/kg for specified fruit juice categories [18]. Traditional fruit-based molasses is produced by concentrating the extracted juice, generally through boiling [1]. The relevance of this value to sugar beet molasses is more limited because sugar beet is not a fruit-derived raw material and the fruit-juice-based value was not established for sugar beet products. Nevertheless, the same value was applied to sugar beet molasses solely to provide a uniform descriptive reference across the four product groups. This application does not imply that the fruit-juice maximum level is legally or toxicologically applicable to sugar beet molasses. Because no product-specific maximum level has been established for patulin in molasses, the proportion of samples above this contextual comparison value should not be interpreted as a rate of regulatory non-compliance. Among the closest available studies of traditional concentrated apple products, Şen (2023) reported that 19 of 26 (73.0%) traditionally produced apple molasses samples exceeded 50 µg/kg [22], while İçli (2019) found patulin concentrations at or above 50 µg/kg in 37 of 39 (94.9%) apple sour samples [21]. These findings provide relevant context for the proportion of 83.3% observed in the apple molasses group of the present study.

Although the proportions above the contextual comparison value ranged numerically from 33.3% to 83.3% among the molasses types, no statistically significant between-group difference was detected. The wide confidence intervals indicate imprecise type-specific estimates, and the small group sizes limit the ability to detect between-group differences. These results do not establish equivalent proportions above the contextual comparison value across molasses types. These proportions describe the fraction of the analyzed samples with concentrations above the selected comparison value and should not be interpreted as direct measures of dietary exposure or health risk.

### 3.5. Associations of Patulin Concentration with pH and °Brix

**Figure 3:**
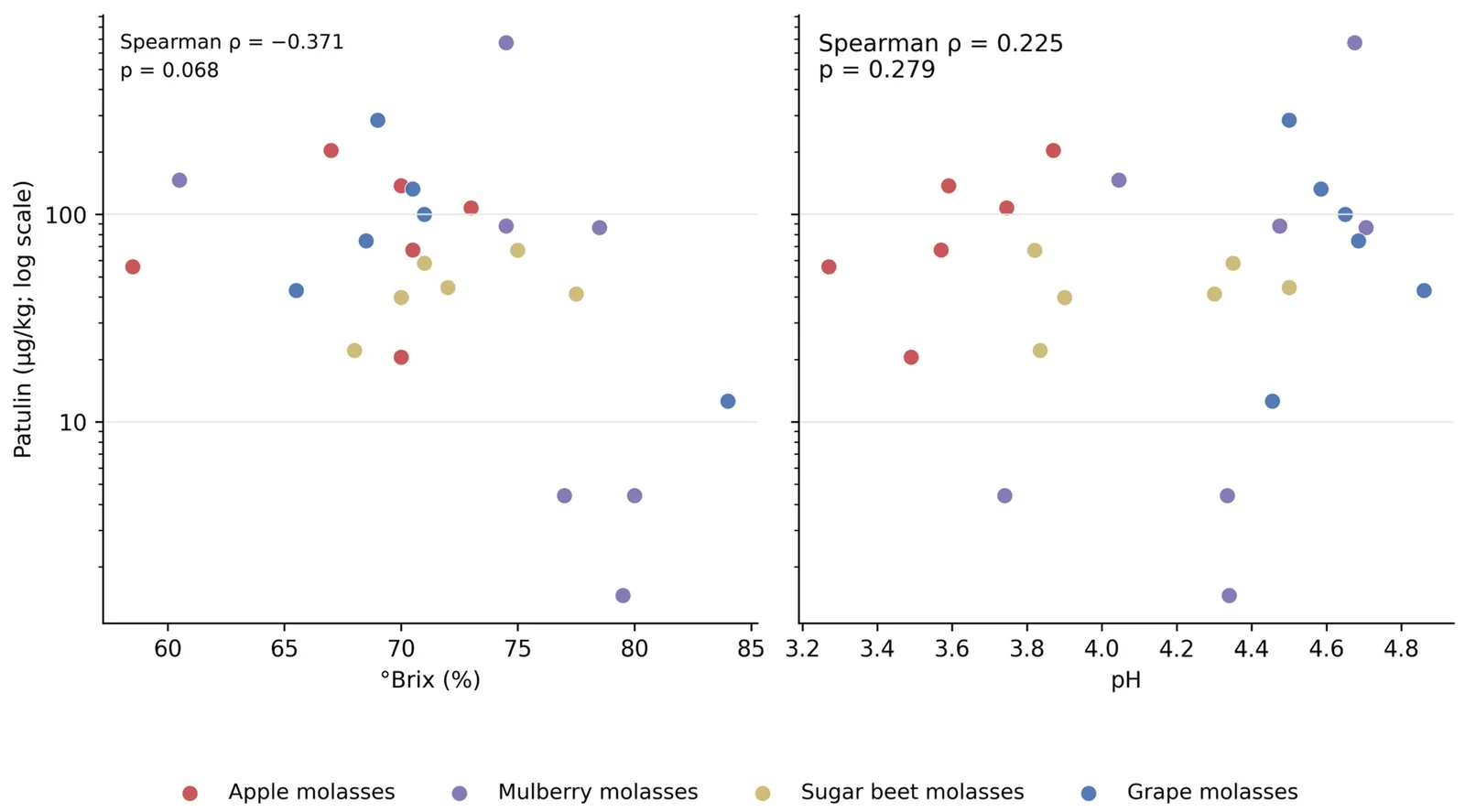
Associations of patulin concentration with °Brix and pH. Points represent individual samples, and colors indicate molasses type. For visualization and correlation analysis, the result below the limit of detection (LOD) was replaced with LOD/2, and the two results between the LOD and the limit of quantification (LOQ) were replaced with LOQ/2. The patulin axis is presented on a logarithmic scale. Spearman’s rank correlation coefficients (ρ) and corresponding *p*-values are displayed in each panel.

No statistically significant correlation was detected between patulin concentration and pH (Spearman’s ρ = 0.225, *p* = 0.279). A negative association was observed between patulin concentration and °Brix, but it was not statistically significant (Spearman’s ρ = −0.371, *p* = 0.068) (Figure 3). The direction and statistical interpretation of both associations remained unchanged in the lower- and upper-bound sensitivity analyses.

In the present study, no statistically significant correlation between final-product pH and patulin concentration was detected in the analyzed sample set. İçli (2019) similarly found no significant relationships between patulin and the chemical properties evaluated in 39 traditionally produced apple sour samples; the reported correlation coefficients were *r* = −0.114 (*p* = 0.489) for pH and *r* = −0.107 (*p* = 0.518) for °Brix [21]. In contrast, Kayisoglu et al. (2026) reported a strong positive correlation between patulin concentration and pH (*r* = 0.78, *p* < 0.01) and a strong negative correlation between patulin concentration and °Brix (*r* = −0.88, *p* < 0.01) in 40 pomegranate molasses samples collected from commercial production batches at a single processing facility [39]. The Spearman coefficient for patulin and °Brix in the present study was also negative, but no statistically significant association was detected.

Experimental evidence from *P. expansum* models suggests that low pH alone may not account for patulin accumulation. Barad et al. (2014) reported that patulin accumulation was associated with dynamic gluconic acid production and the accompanying acidification process rather than merely with low pH or the final pH of the medium [20]. These acidification dynamics were not assessed in the present study. Given the limited sample size, the absence of statistically significant correlations does not rule out potentially meaningful associations and cannot establish the influence of pH or soluble-solids content on patulin occurrence during production.

### 3.6. Screening-Level Dietary Exposure to Patulin

Under the serving-based screening scenario of 27 g of molasses per day for a 70-kg adult, the EDI calculated using the overall median patulin concentration was 0.0260 µg/kg body weight/day. This scenario was based on a standard serving rather than observed habitual consumption and should not be interpreted as a population-level dietary exposure estimate. This corresponded to an HQ of 0.065 and 6.5% of the PMTDI of 0.4 µg/kg body weight/day established by the Joint Food and Agriculture Organization of the United Nations (FAO)/World Health Organization (WHO) Expert Committee on Food Additives (JECFA) [17].

Based on the molasses-type-specific median concentrations, the estimated EDI ranged from 0.0166 µg/kg body weight/day for sugar beet molasses to 0.0338 µg/kg body weight/day for apple molasses. The corresponding HQ values ranged from 0.041 to 0.084, representing 4.1–8.4% of the PMTDI.

In the highest-concentration scenario, based on the maximum observed patulin concentration of 672.50 µg/kg, the EDI was 0.2594 µg/kg body weight/day. This corresponded to an HQ of 0.648 and 64.8% of the PMTDI. Under the assumptions used, all evaluated adult scenarios yielded HQ values below 1. The highest-concentration calculation represents a conservative hypothetical scenario in which a 70 kg adult consumes a 27 g serving of the sample with the highest measured patulin concentration every day; it should not be interpreted as an estimate of typical consumer exposure (Table 4). The exposure estimates remained unchanged in the lower- and upper-bound sensitivity analyses because the overall median, molasses-type-specific medians, and maximum concentration used in the calculations were unchanged.

Although all calculated HQ values were below 1 under the assumptions used, the maximum-concentration scenario accounted for 64.8% of the PMTDI from the assumed daily consumption of a single 27-g serving of molasses. This scenario assumes repeated consumption of the sample with the highest observed patulin concentration and therefore should not be interpreted as representing typical exposure. Moreover, the assessment included patulin exposure from molasses alone and did not account for exposure from other dietary sources. Accordingly, HQ values below 1 in the evaluated scenarios should not be interpreted as a comprehensive demonstration of dietary safety.

Among the studies identified, Kayisoglu et al. (2026) provided the most directly comparable dietary exposure assessment [39]. Using 40 pomegranate molasses samples produced in a commercial processing facility, the authors estimated mean adult patulin intakes of 0.0052–0.0053 and 0.0104–0.0106 µg/kg bw/day under hypothetical consumption scenarios of 5 and 10 g/day, respectively. The corresponding mean HQ values were 0.0132 and 0.0264. These estimates were numerically lower than the overall-median estimate obtained in the present study. However, Kayisoglu et al. evaluated a different molasses matrix, used mean rather than median patulin concentrations, and assumed lower daily consumption amounts. Therefore, the numerical difference should not be interpreted as evidence of a difference in dietary exposure between molasses types. The same study reported slightly higher exposure estimates for children than for adults under its age-specific assumptions.

The exposure estimates in the present study depend directly on the assumed consumption amount. The 27-g amount specified in TÜBER 2022 represents a standard serving rather than observed habitual daily intake. Accordingly, the calculated values represent serving-based screening scenarios for a 70-kg adult rather than population-level dietary exposure estimates. The assessment was restricted to adults because reliable age-specific data on habitual molasses consumption among children were unavailable. Therefore, the resulting estimates apply only to the specified adult scenario and should not be extrapolated to children.

## 4. Limitations

The findings should be interpreted in light of several limitations. First, this exploratory study included a limited number of purposively selected samples (*n* = 25; 6–7 samples per product type) obtained from household producers and sales points offering homemade regional products in selected provinces. Therefore, the findings describe only the analyzed sample set and should not be considered representative of traditional molasses production throughout Türkiye. In particular, the observed proportion of samples above the 50 µg/kg contextual comparison value should not be interpreted as a nationally representative estimate. The small group sizes and substantial within-group variability, including the high maximum concentration observed in mulberry molasses, also limit the precision of the between-group comparisons. Accordingly, the near-zero epsilon-squared estimate for patulin should be interpreted cautiously and should not be considered evidence of an absence of differences among molasses types.

Second, product type and geographical origin were partly confounded. Consequently, the contribution of raw-material type to the between-group comparisons could not be distinguished from possible regional influences or differences in raw-material characteristics, pre-collection storage conditions, and production practices. This limits the interpretation of both the observed pH differences and the absence of statistically significant differences in patulin concentrations and °Brix.

Third, each producer was represented by a single sample, and seasonal and production-batch variability were not evaluated. Exact production dates, individual collection dates, and sample-specific storage durations were also unavailable. Because sample collection extended over approximately six months and the analyses were conducted after the final sample had been obtained, the duration of laboratory storage differed among samples. The possible effects of differences in production and storage history on patulin concentrations could not therefore be determined.

Fourth, method performance was not established across all four molasses matrices. Recovery and repeatability were evaluated only in an apple-derived molasses matrix, while the FAPAS apple-juice proficiency-test material was used solely as an external quality-control material. Although the same extraction and HPLC-DAD procedure was applied to all samples, this does not demonstrate equivalent analytical performance across different matrices. No matrix-specific recovery or repeatability studies, matrix-matched calibration, or standard-addition experiments were performed for mulberry, grape, or sugar beet molasses. Given the reported compositional differences among molasses types [3,4], matrix-dependent effects on extraction recovery and analytical response cannot be excluded, and the magnitude and direction of any resulting bias remain unknown. This uncertainty extends to the reported concentrations, the proportions of samples above the contextual comparison value, and the derived dietary exposure estimates. Accordingly, quantitative results involving the non-apple matrices and comparisons across molasses types should be considered preliminary until supported by matrix-specific validation.

Finally, the dietary exposure assessment was based on a 27 g standard serving rather than nationally representative data on habitual molasses consumption. The assessment was limited to adults and did not characterize exposure among children, who may receive a higher body-weight-adjusted dose under comparable consumption conditions. The resulting estimates should therefore be interpreted as serving-based screening scenarios and not as population-level exposure estimates or comprehensive assessments of health risk.

Future studies should include larger and geographically representative sample sets, multiple seasons and production batches from individual producers, matrix-specific analytical validation for each molasses type, and population-based consumption data for both adults and children.

## 5. Conclusions

This exploratory study compared patulin concentrations, pH, and soluble-solids content (°Brix) in 25 purposively selected samples of traditionally produced apple, mulberry, sugar beet, and grape molasses from selected provinces in Türkiye, with 6–7 samples per product type. Patulin was detected in at least one sample from each molasses type. Within the analyzed sample set, no statistically significant differences in patulin concentrations or °Brix were detected among the four molasses types, whereas apple molasses had a significantly lower pH than mulberry and grape molasses. No statistically significant associations were detected between patulin concentration and either final-product pH or °Brix. The absence of statistically significant differences in patulin concentrations and °Brix does not establish equivalence among molasses types; these comparisons remain exploratory because of the small group sizes and the partial confounding between product type and geographical origin. Moreover, because matrix-specific recovery and precision were not established for mulberry, grape, or sugar beet molasses, quantitative findings involving these matrices should be considered preliminary.

Fifteen of the 25 samples had patulin concentrations above the 50 µg/kg contextual comparison value. This descriptive finding should not be interpreted as evidence of regulatory non-compliance because no product-specific maximum level has been established for patulin in molasses. In the serving-based adult exposure assessment, median-based HQ values ranged from 0.041 to 0.084, whereas the maximum-concentration scenario yielded an HQ of 0.648. Under the assumptions used, all evaluated HQ values were below 1. However, the maximum-concentration calculation represents a conservative hypothetical scenario based on the daily consumption of the sample with the highest measured patulin concentration. Because the assessment assumed a standard 27-g serving for a 70-kg adult rather than observed habitual consumption and excluded other dietary sources of patulin, the estimates should not be interpreted as population-level exposure estimates or comprehensive assessments of health risk and should not be extrapolated to children.

To the best of our knowledge, this is the first study to compare patulin occurrence in these four traditional molasses types within a single research design. These preliminary findings describe the analyzed sample set and do not establish the prevalence or geographical distribution of patulin in traditional molasses in Türkiye. Nevertheless, they provide initial evidence supporting the need for broader investigation of patulin occurrence in these products. Future studies should use larger and geographically representative sample sets; include multiple producers, seasons, and production batches; validate the analytical method separately for each molasses matrix; compare traditionally and commercially produced products; and incorporate habitual-consumption data for both adults and children.

## Figures and Tables

**Table 1:** Analytical results for the FAPAS surplus test material from Proficiency Test 1632 017.

Replicate	Measured Concentration (µg/L)	Assigned Concentration (µg/L)	Recovery (%)
1	40.80	37.50	108.80
2	40.00	37.50	106.67
3	40.00	37.50	106.67
4	41.00	37.50	109.33
5	39.20	37.50	104.53
6	40.04	37.50	106.77
Mean ± SD	40.17 ± 0.65	37.50	107.13 ± 1.73

Note. Recovery was calculated as the measured concentration divided by the assigned concentration and multiplied by 100. The material was analyzed as six independent preparations outside the original proficiency-testing round and was used solely for analytical quality-control purposes. Therefore, the results should not be interpreted as a formal proficiency-test score. FAPAS, Food Analysis Performance Assessment Scheme; RSD, relative standard deviation; SD, standard deviation.

**Table 2:** Physicochemical characteristics and patulin concentrations according to molasses type.

Variable	Apple Molasses (*n* = 6)	Mulberry Molasses (*n* = 7)	Sugar Beet Molasses (*n* = 6)	Grape Molasses (*n* = 6)	*p*-Value	ε^2^
**pH**	3.58 ^a^ (3.51–3.71) [3.27–3.87]	4.34 ^b^ (4.19–4.58) [3.74–4.71]	4.10 ^ab^ (3.85–4.34) [3.82–4.50]	4.62 ^b^ (4.52–4.68) [4.46–4.86]	0.001	0.61
**Soluble solids (°Brix)**	70.0 (67.8–70.4) [58.5–73.0]	77.0 (74.5–79.0) [60.5–80.0]	71.5 (70.2–74.2) [68.0–77.5]	69.8 (68.6–70.9) [65.5–84.0]	0.108	0.15
**Patulin (µg/kg)**	87.58 (58.80–130.08) [20.55–203.60]	86.45 (4.41–117.15) [<LOD–672.50]	42.93 (40.16–54.76) [22.10–67.30]	87.38 (50.85–124.61) [12.60–284.80]	0.442	0.00

Note. Data are presented as median (Q1–Q3) [minimum–maximum]. For the statistical analyses of patulin, the result below the LOD was replaced with LOD/2, whereas the two results between the LOD and LOQ were replaced with LOQ/2. Accordingly, the first quartile reported for mulberry molasses reflects the substituted values, whereas its minimum is reported as <LOD rather than as an exact concentration. Differences among molasses types were evaluated using the Kruskal–Wallis test. Epsilon-squared (ε^2^) was reported as the effect-size measure. For pH, pairwise comparisons were performed using Dunn’s test with Holm adjustment. Values within the pH row that do not share a superscript letter differ significantly (adjusted *p* < 0.05). No pairwise comparisons were performed for °Brix or patulin because the corresponding omnibus tests were not statistically significant. LOD, limit of detection; LOQ, limit of quantification; Q1, first quartile; Q3, third quartile.

**Table 3:** Numbers and proportions of samples with patulin concentrations above the 50 µg/kg contextual comparison value.

Molasses Type	Total Samples, *n*	Samples Above the Contextual Comparison Value, *n*	Proportion, (%)	Exact 95% CI (%)
**Apple molasses**	6	5	83.3	35.9–99.6
**Mulberry molasses**	7	4	57.1	18.4–90.1
**Sugar beet molasses**	6	2	33.3	4.3–77.7
**Grape molasses**	6	4	66.7	22.3–95.7
**Overall**	25	15	60.0	38.7–78.9

Note. CI, confidence interval. Exact 95% confidence intervals were calculated using the Clopper–Pearson method. Differences among molasses types were evaluated using the Fisher–Freeman–Halton exact test (*p* = 0.429). The value of 50 µg/kg was used solely as a contextual comparison value and should not be interpreted as a product-specific regulatory limit or safety threshold for molasses.

**Table 4:** Serving-based screening-level dietary exposure to patulin in adult consumers.

Patulin Concentration Scenario	Patulin Concentration (µg/kg)	EDI (µg/kg *BW*/Day)	HQ	Contribution to PMTDI (%)
**Overall median**	67.30	0.0260	0.065	6.5
**Apple molasses median**	87.58	0.0338	0.084	8.4
**Mulberry molasses median**	86.45	0.0333	0.083	8.3
**Sugar beet molasses median**	42.93	0.0166	0.041	4.1
**Grape molasses median**	87.38	0.0337	0.084	8.4
**Maximum observed concentration**	672.50	0.2594	0.648	64.8

Note. *BW*, body weight; EDI, estimated daily intake; HQ, hazard quotient; PMTDI, provisional maximum tolerable daily intake. EDI was calculated as (C × IR)/BW, assuming daily consumption of one standard serving of molasses (*IR* = 0.027 kg/day), as specified in the Türkiye Beslenme Rehberi (TÜBER) 2022 [29], and a default adult body weight (*BW*) of 70 kg [30]. HQ was calculated by dividing the EDI by the JECFA PMTDI [17] for patulin (0.4 µg/kg bw/day). The 27-g value represents a standard serving size rather than nationally observed habitual daily consumption; therefore, the estimates should be interpreted as serving-based screening scenarios rather than population-level dietary exposure estimates.

## Data Availability

The original contributions presented in this study are included in the article and supplementary material. Further inquiries can be directed to the corresponding author.

## Supplementary Materials

The following supporting information can be downloaded at: https://www.mdpi.com/article/10.3390/foods15213798/s1, Table S1: Back-calculated concentrations and residuals for the six-level external-standard calibration; Figure S1: Overlaid chromatograms of the six patulin calibration standards (0.050–2.000 µg/mL) recorded at 276 nm; Figure S2: Representative chromatograms of (A) the lowest patulin calibration standard at 0.050 µg/mL and (B) a mixed standard solution containing 0.050 µg/mL patulin and 5 µg/mL 5-hydroxymethylfurfural (HMF); Figure S3: Representative chromatograms obtained during assessment of the apple-derived molasses matrix; Figure S4: Representative chromatograms of patulin-containing molasses samples.

## Abbreviations

The following abbreviations are used in this manuscript:

DAD	Diode-array detector
HMF	5-Hydroxymethylfurfural
HPLC-DAD	High-performance liquid chromatography with diode-array detection
HPLC-UV	High-performance liquid chromatography with ultraviolet detection
LC–MS	Liquid chromatography–mass spectrometry
FAO	Food and Agriculture Organization of the United Nations
WHO	World Health Organization
JECFA	Joint FAO/WHO Expert Committee on Food Additives
PMTDI	Provisional maximum tolerable daily intake
LOD	Limit of detection
LOQ	Limit of quantification
RSD	Relative standard deviation
FAPAS	Food Analysis Performance Assessment Scheme
TÜBER	Türkiye Beslenme Rehberi
EDI	Estimated daily intake
HQ	Hazard quotient
IR	Intake rate
BW	Body weight
CI	Confidence interval
SD	Standard deviation
