# Supplementary material for: Patulin Occurrence in Traditional Turkish Molasses (Pekmez): Comparisons Across Product Types and Associations with pH and Soluble Solids
Source: Foods. 2026 Oct 23;15(21):3798. doi: 10.3390/foods15213798 (PMC13762773; doi:10.3390/foods15213798)
Supplement: Supplementary file 1 [file foods-15-03798-s001.zip › foods-4629386-supplementary.pdf]

Supplementary Materials

Patulin Occurrence in Traditional Turkish Molasses (Pekmez): Comparisons Across Product Types and Associations with pH and Soluble Solids

Table S1. Back-calculated concentrations and residuals for the six-level external-standard calibration.

Nominal concentration (µg/mL)	Peak area	Back-calculated concentration (µg/mL)	Residual (µg/mL)	Relative error (%)
0.050	8576	0.053494	+0.003494	+6.99
0.100	17582	0.103641	+0.003641	+3.64
0.250	43070	0.245564	-0.004436	-1.77
0.500	87811	0.494691	-0.005309	-1.06
1.000	178933	1.002076	+0.002076	+0.21
2.000	358248	2.000539	+0.000539	+0.03

Note. Back-calculated concentration was obtained from $C = (5.568202 \times 10^{-6})A + 5.741274 \times 10^{-3}$, where C is patulin concentration and A is peak area. Residual was calculated as back-calculated minus nominal concentration, and relative error as residual divided by nominal concentration × 100. One response was available at each calibration level; therefore, formal lack-of-fit and heteroscedasticity assessments were not performed.

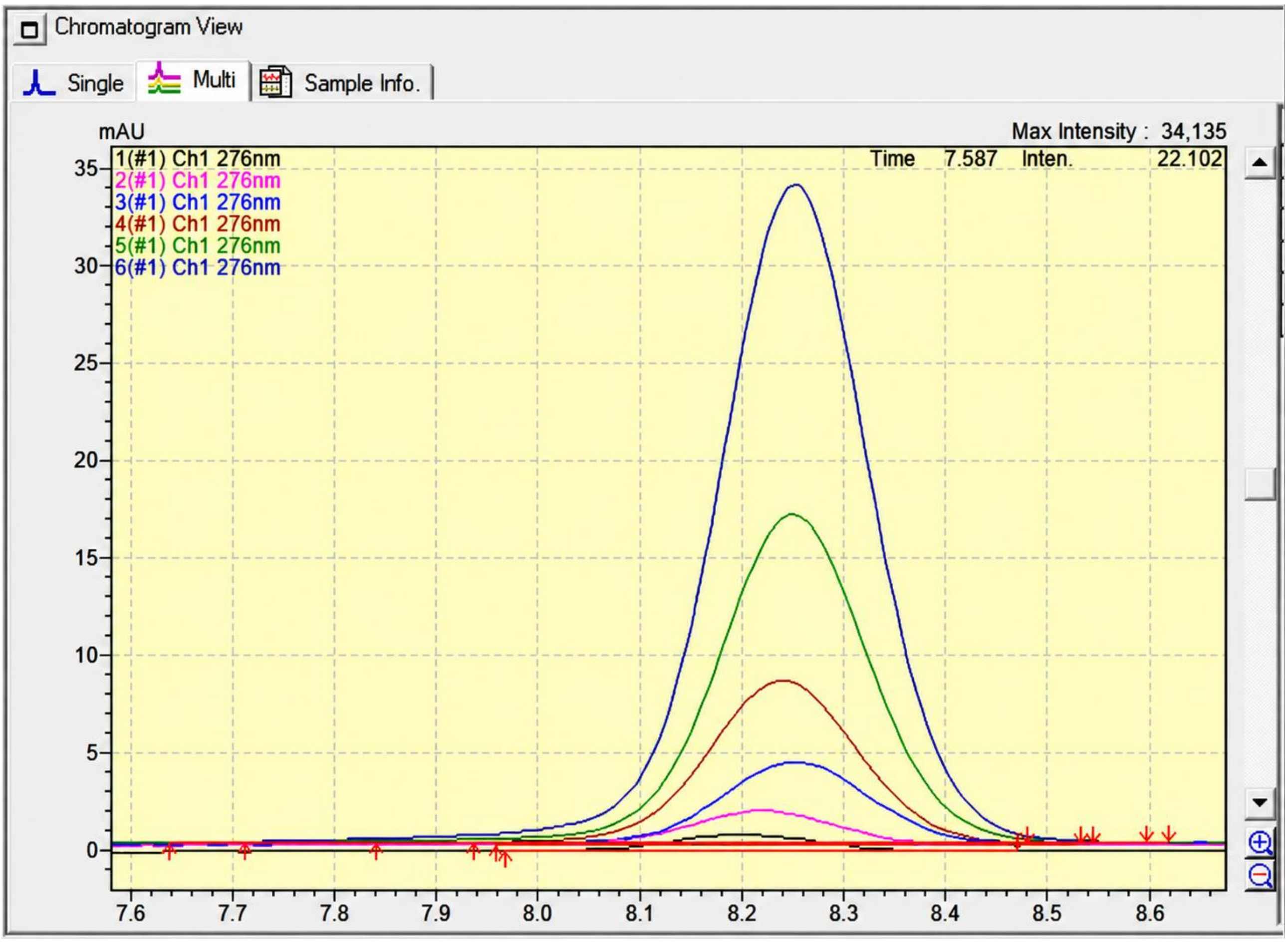

Figure S1. Overlaid chromatograms of the six patulin calibration standards (0.050–2.000 µg/mL) recorded at 276 nm.

(A) Patulin standard, 0.050 µg/mL

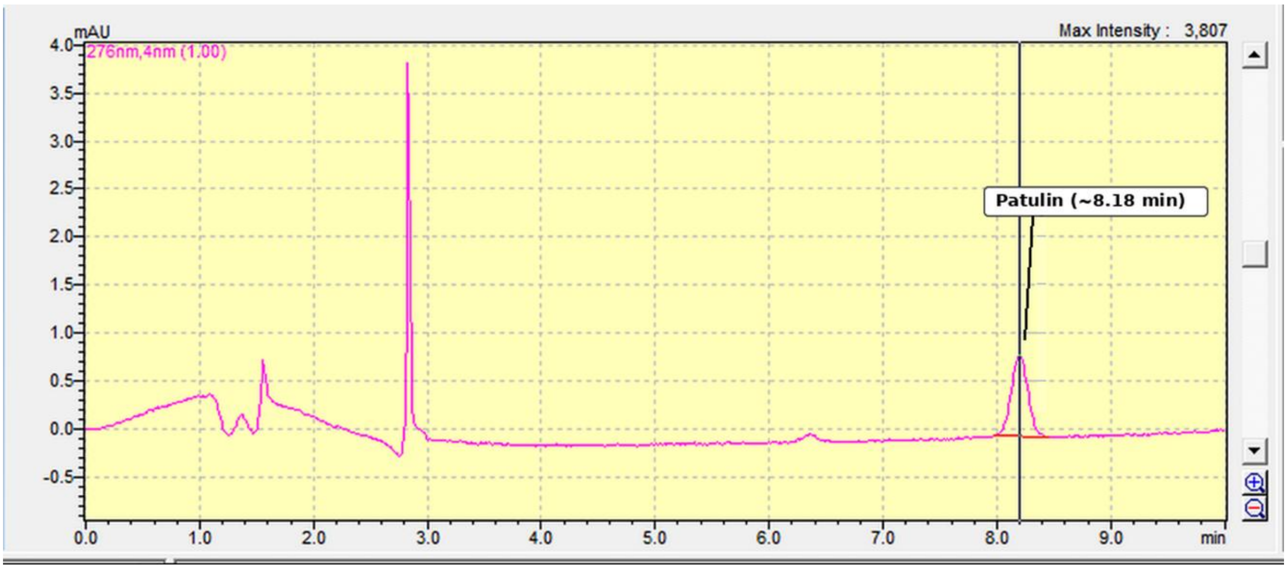

(B) Mixed standard containing patulin and HMF

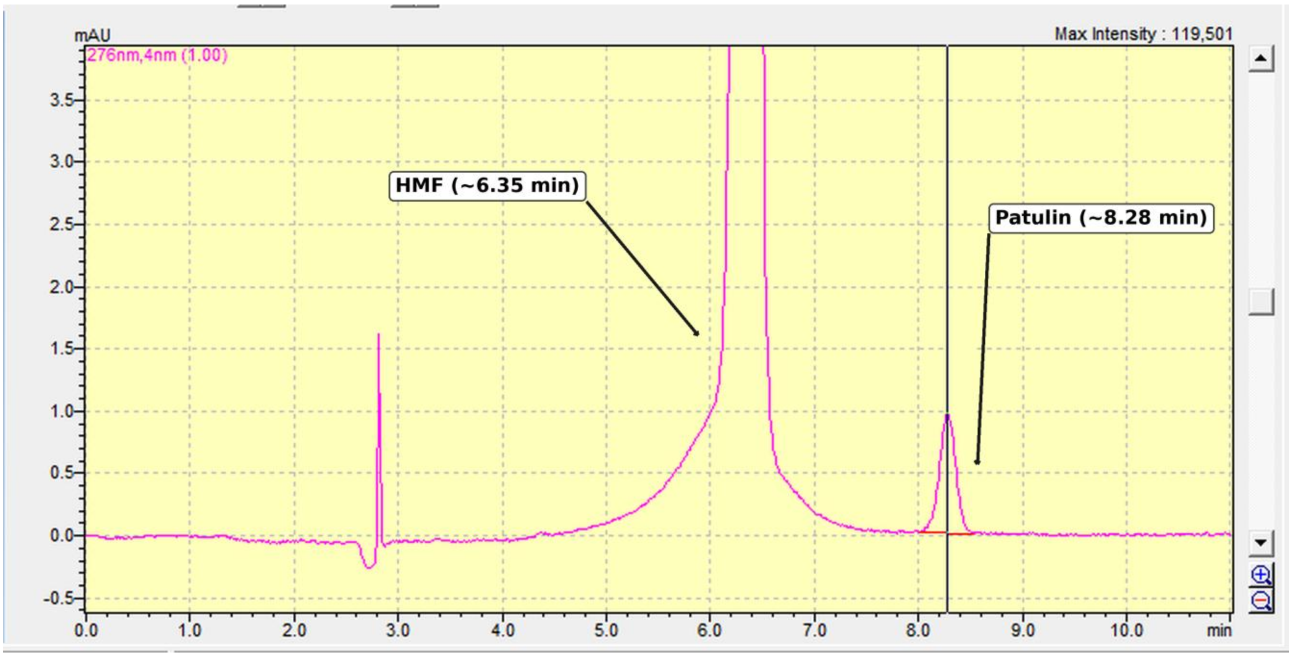

Figure S2. Representative chromatograms of (A) the lowest patulin calibration standard at 0.050 µg/mL and (B) a mixed standard solution containing 0.050 µg/mL patulin and 5 µg/mL 5-hydroxymethylfurfural (HMF). Patulin eluted at approximately 8.18 min in panel A. In the mixed standard shown in panel B, HMF and patulin eluted at approximately 6.35 and 8.28 min, respectively, demonstrating their chromatographic separation under the specified standard-solution conditions.

(A) Apple-derived matrix blank

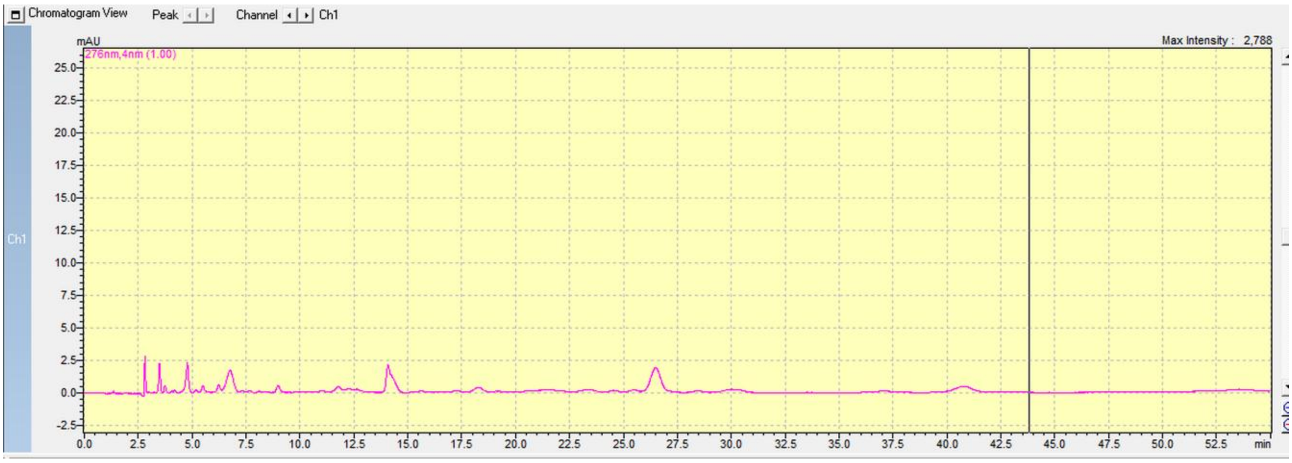

(B) Fortified apple-derived matrix

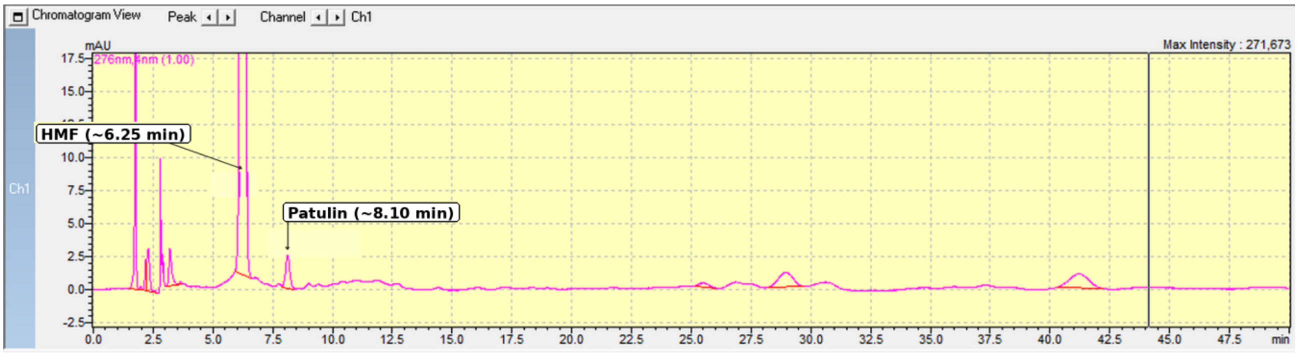

(C) Enlarged HMF and patulin retention region

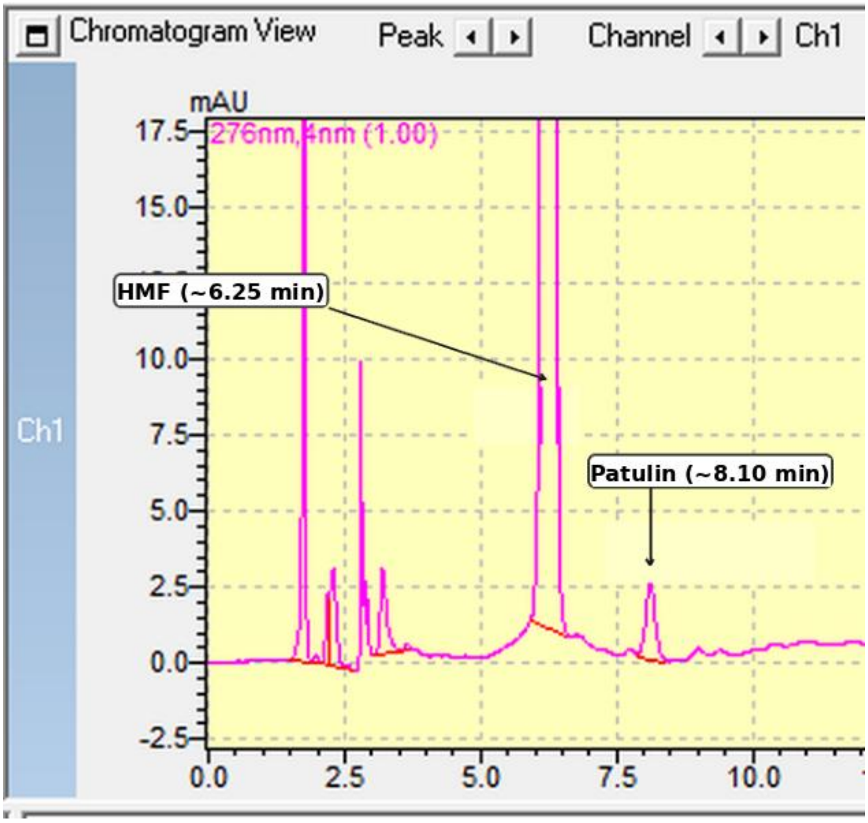

Figure S3. Representative chromatograms obtained during assessment of the apple-derived molasses matrix. (A) Apple-derived matrix blank subjected to the complete sample-preparation and extraction procedure; no detectable peaks were observed at the retention times of HMF or patulin under the applied analytical conditions. (B) A separately prepared

fortified matrix sample obtained by adding 1 mL of an aqueous working solution containing 0.15 µg/mL patulin and 12.5 µg/mL HMF to 5 g of the blank matrix before the complete extraction procedure. The patulin addition corresponded to a sample-equivalent fortification level of 30 µg/kg. The final extract was reconstituted to 1 mL. (C) Enlarged chromatographic region showing the separation of HMF and patulin in the fortified matrix. This separately prepared sample was used to illustrate chromatographic behavior and the separation of HMF and patulin and was not one of the replicates used to calculate the recovery and precision values reported in Section 2.6.

(A) Mulberry molasses, 672.50 µg/kg

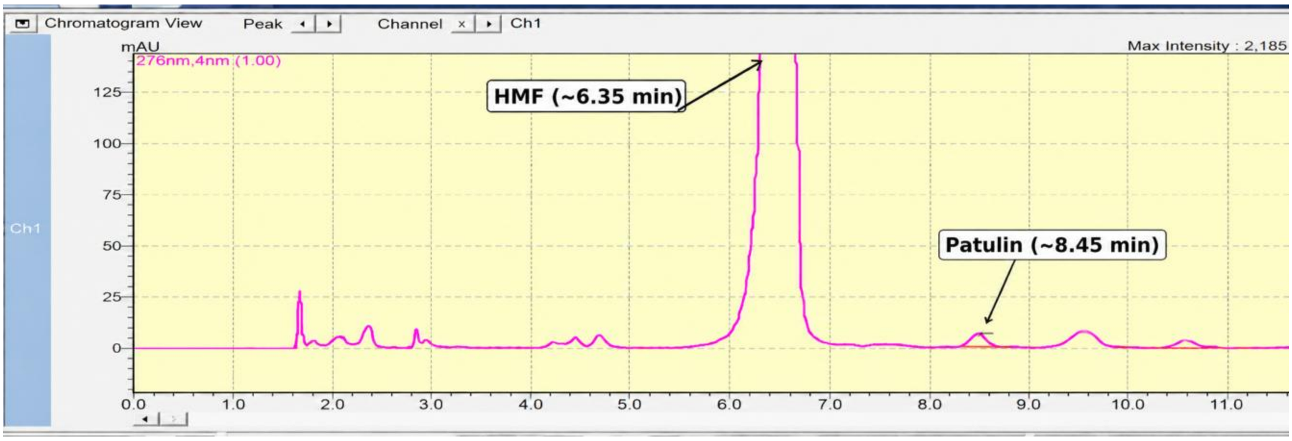

(B) Apple molasses, 203.60 µg/kg

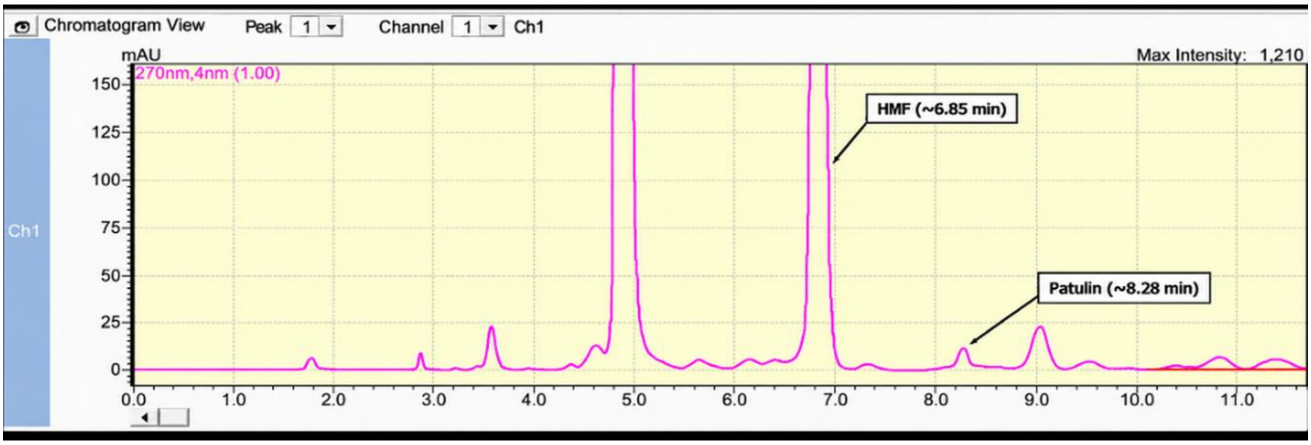

(C) Sugar beet molasses, 67.30 µg/kg

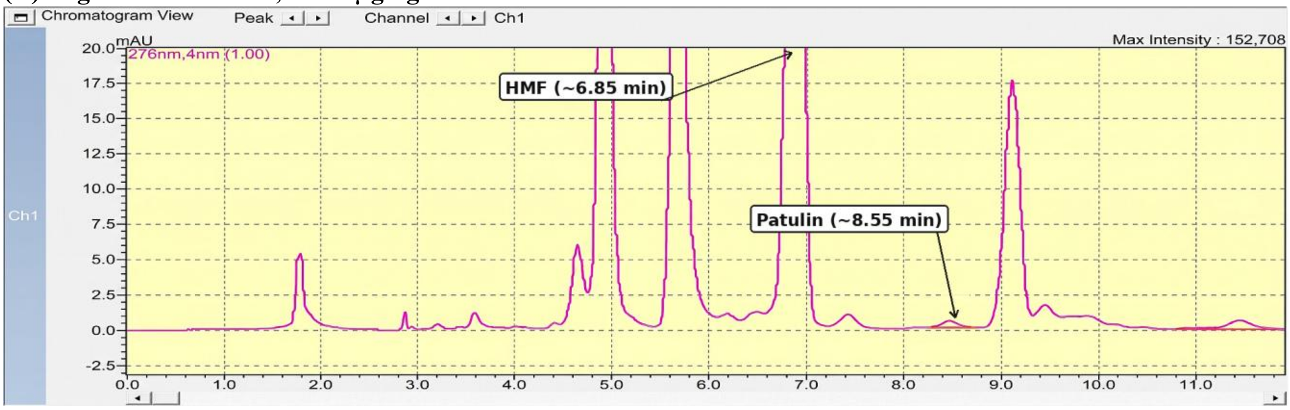

(D) Grape molasses, 284.80 µg/kg

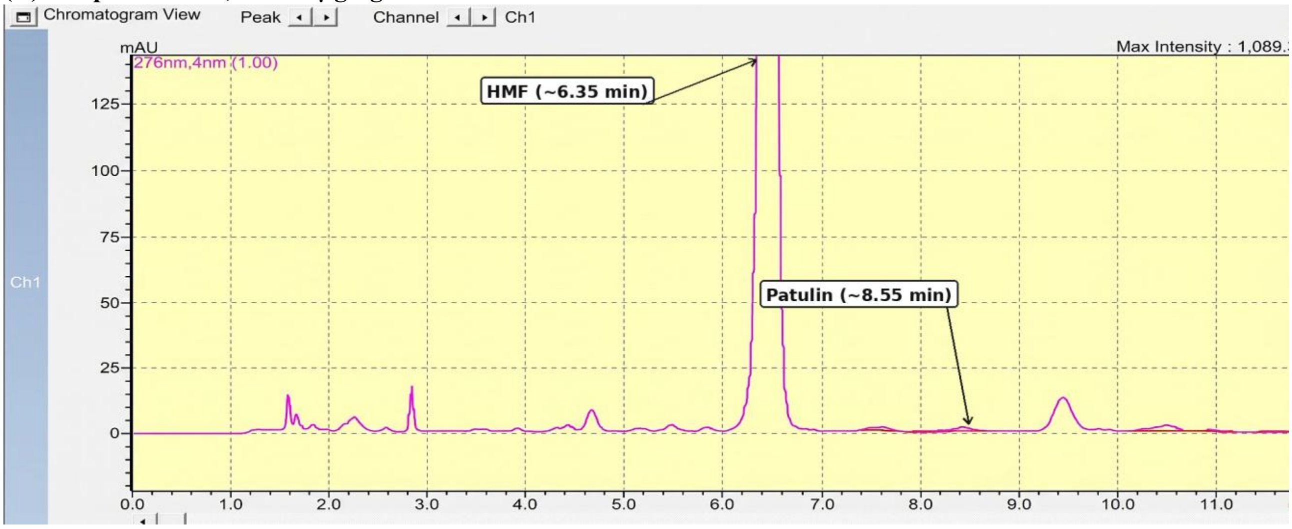

Figure S4. Representative chromatograms of patulin-containing molasses samples. (A) Mulberry molasses sample with the highest measured patulin concentration; (B) apple molasses sample with the highest measured concentration within the apple-molasses group; (C) sugar beet molasses sample with the highest measured concentration within the sugar-beet-molasses group; and (D) grape molasses sample with the highest measured concentration within the grape-molasses group. Sample chromatographic runs were extended to 55 min to minimize carryover from late-eluting matrix constituents; the chromatograms shown here were cropped to approximately 11–12 min to display the HMF and patulin peak regions more clearly. Peak assignment was based on retention-time agreement with the patulin reference standard. These chromatograms provide representative chromatographic evidence but do not constitute definitive spectral confirmation of peak identity or exclude all possible matrix-specific co-elution. The observed retention times of the labelled HMF and patulin peaks are provided separately in each panel; minor retention-time variation was observed among chromatographic runs.
